# Autosomal Dominant Missense *DAG1* Variant Linked to Mild–Moderate LGMD R16

**DOI:** 10.1155/humu/7451586

**Published:** 2026-07-04

**Authors:** Edoardo Malfatti, Alexandru Caramizaru, Federica Trentin, Andreea Dumitrescu, Luca Sali, Alexandra Bastian, Hane Lee, Homa Tajsharghi, Camille Verebi, Juliette Nectoux, France Leturcq, Rahul Phadke, Anna Sarkozy, Adnan Manzur, Redouane Fodil, Amelia Dobrescu

**Affiliations:** ^1^ Reference Center for Neuromuscular Disorders, APHP Henri Mondor University Hospital, Créteil, France; ^2^ University Paris Est Créteil, Inserm U955, IMRB, Créteil, France, inserm.fr; ^3^ Regional Center for Medical Genetics Dolj, Craiova, Romania; ^4^ Department of Medical Genetics, University of Medicine and Pharmacy of Craiova, Craiova, Romania, umfcv.ro; ^5^ Department of Medical and Surgical Sciences (DIMEC), University of Bologna, Bologna, Italy, unibo.it; ^6^ Department of Child and Infancy Neuropsychiatry, Neuromuscular Disorders, IRCCS Institute for Treatment and Research, The Institute of Neurological Sciences of Bologna, Bologna, Italy; ^7^ Department of Pathology, Colentina Clinical Hospital, Bucharest, Romania, spitalul-colentina.ro; ^8^ 3billion Inc., Seoul, South Korea; ^9^ School of Health Sciences, Division of Biomedicine, University of Skovde, Skovde, Sweden; ^10^ Service de Médecine Génomique des Maladies de Système et d′Organe, Fédération de Génétique et de Médecine Génomique, APHP.Centre-Université Paris Cité, Hôpital Cochin, Paris, France, aphp.fr; ^11^ Dubowitz Neuromuscular Centre, Great Ormond Street Hospital, London, England, UK, nhs.uk

**Keywords:** alpha-dystroglycan, *DAG1*, glycosylation, heterozygous, LGMD R16, mild–moderate phenotype, missense

## Abstract

Limb‐girdle muscular dystrophies (LGMDs) are disorders with an important clinical heterogeneity, usually involving proximal limb muscles. One subtype, LGMD R16 (LGMD 2P), is an autosomal recessive condition caused by pathogenic variants in *DAG1*, with clinical presentations ranging from mild to extremely severe forms. *DAG1* is responsible for producing dystroglycan, an essential complex in the muscular protein network. Following translation, dystroglycan is cleaved into alpha‐dystroglycan, which undergoes glycosylation and acts as a sarcolemmal receptor for extracellular proteins, and beta‐dystroglycan, which connects to dystrophin. In recent years, heterozygous nonsense or frameshift *DAG1* variants have been linked with asymptomatic hyperCKemia (increased serum creatine kinase levels) or mild muscular phenotypes characterized by fatigability and myalgia. Here, we describe a Romanian family comprising four affected individuals (one father and three sons) carrying the heterozygous missense *DAG1* variant NM_004393.6:c.887G>A, NP_004384.5:p.(Gly296Asp) and showing a mild–moderate muscular phenotype similar to previous cases of *DAG1* haploinsufficiency. Three of the affected individuals exhibit myopathic changes in muscle biopsies (increased fiber size variability, internalized nuclei, and regenerating fibers), while two demonstrate reduced alpha‐dystroglycan glycosylation in muscle tissue. Atomic force microscopy findings in myoblasts from one patient showed a significantly lower stiffness compared to controls. These findings align with prior reports and further support the pathogenicity of this variant.

## 1. Introduction

Limb‐girdle muscular dystrophies (LGMDs) are a clinically and genetically heterogeneous group of entities usually characterized by progressive proximal limb muscle deterioration. Other muscle involvement is possible, and the presentation can range from severe, early‐onset, and rapidly progressive forms to a mild, nonimpairing symptomatology [1]. The mode of inheritance can be autosomal dominant or recessive, with the latter being more common [2, 3].

LGMD R16 (formerly classified as LGMD 2P) is classically a rare autosomal recessive LGMD subtype determined by pathogenic *DAG1* (OMIM 128239) variants [1, 4, 5], a gene encoding dystroglycan. After translation, the dystroglycan complex is divided into two subunits: alpha‐dystroglycan, present in the sarcolemma (acting as a laminin and perlecan receptor), and beta‐dystroglycan, which crosses the cellular membrane and interacts with dystrophin [6]. Alpha‐dystroglycan interacts with LARGE1 (like‐acetylglucosaminyltransferase‐1) and contributes to the production of matriglycan, a polysaccharide that binds to extracellular matrix (ECM) proteins such as laminin; in order to function properly, alpha‐dystroglycan needs to undergo important post‐translational processes and glycosylation [7, 8]. Disorders involving reduced alpha‐dystroglycan glycosylation are also known as dystroglycanopathies [9, 10].

Clinical manifestations of LGMD R16 include fatigability, proximal muscle weakness, motor difficulties, and elevated creatine kinase (CK) levels, usually with an early‐onset and a slow progression; intellectual disability can also be present, differentiating this disorder from other LGMDs [11, 12]. Moreover, biallelic variants in *DAG1* can also lead to a significantly more severe muscle–eye–brain disease‐like phenotype with muscular dystrophy, brain and eye malformations, and profound intellectual disability [13, 14].

Recently, heterozygous frameshift or nonsense *DAG1* variants have been linked to a mild, asymptomatic, or paucisymptomatic muscular phenotype and hyperCKemia, expanding the clinical and genetic spectrum of LGMD R16 and introducing *DAG1* haploinsufficiency as a possible cause for an extremely mild phenotype [15, 16]. In this paper, we report the first pathogenic heterozygous missense *DAG1* variant, NM_004393.6:c.887G>A, NP_004384.5:p.(Gly296Asp), present in four moderately affected, related Romanian patients, further adding to the LGMD R16 landscape.

## 2. Materials and Methods

**Figure 1:**
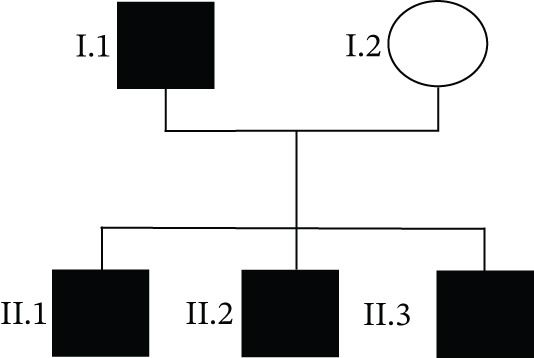
Pedigree. Family tree highlighting affected individuals: Patient I.1—father; Patient II.1—eldest son; Patient II.2—middle son; and Patient II.3—youngest son.

The four patients (PI.1—father, PII.1—eldest son, PII.2—middle son, and PII.3—youngest son) were clinically evaluated in three separate centers: Regional Center for Medical Genetics Dolj (Craiova, Romania); Neuromuscular Diseases Reference Center, APHP Henri Mondor Hospital (Créteil, France); and Great Ormond Street Hospital for Children (GOSH, London, United Kingdom). PII.1 is designated as the proband, being the first person in the family for whom medical attention was sought. A family tree is available in Figure 1.

Muscle biopsies were performed for PII.1 (age 4, left gastrocnemius, Colentina Clinical Hospital, Bucharest, Romania, 2019), PII.2 (age 5, left quadriceps, GOSH, 2021), and PI.1 (age 41, left deltoid, Henri Mondor Hospital, 2024).

The family has undergone extensive genetic testing across multiple laboratories, including initial analysis of the *DMD* gene for PII.1 and PII.2 and whole exome sequencing (WES) for PII.2. Comprehensive testing of the entire family (mother, father, and three sons) was subsequently performed at GOSH, encompassing a rhabdomyolysis/metabolic myopathy panel and a congenital myopathy (CMYO)/muscular dystrophy panel. Additionally, PII.1 underwent evaluation with a muscular dystrophy panel at Cochin Hospital in Paris and WES within the NeuroMyoDredger project [17].

Alpha‐dystroglycan glycosylation analysis (Western blot) was performed for PI.1 and PII.1 with anti‐*α*‐dystroglycan antibody, clone VIA4‐1 (Merck Millipore, Germany), whereas an LGMD gene panel (including *DAG1*) RNA sequencing was conducted for PII.1 (RT‐PCR, capture—Nimblegen, Roche; sequencing—Next Seq550 Illumina; bioinformatics protocol—PolyRNAseq, Polyweb), both at Cochin Hospital, Paris. These investigations were carried out using muscle tissue samples.

The stiffness of PI.1 and control‐derived myoblasts was evaluated by atomic force microscopy (AFM; JPK NanoWizard Sense+, Bruker) using a pyramidal probe calibrated with the thermal noise method [18]. For each cell, multiple indentations were performed over a defined surface area, and force–distance curves were analyzed with a custom MATLAB program. The elastic modulus was calculated according to Bilodeau′s model [19] with correction for local tilt [20]. For each cell, stiffness maps and distributions of elastic moduli were generated. The distribution of Young′s modulus values across individual stiffness maps showed a positive skewness (0.907 ± 0.491), indicating a non‐normal distribution at the pixel level; therefore, the modal value was extracted as a representative measure of cell stiffness for each cell [21]. Group comparison of modal values was performed using the two‐tailed Mann–Whitney *U* test (GraphPad Prism), given the small sample size (*n* = 7 per group). Results are presented as mean ± standard deviation. Differences were considered statistically significant at *p* < 0.05.

## 3. Results

### 3.1. Clinic

None of the four affected individuals had a notable prenatal history. Furthermore, all demonstrated normal neurological development, achieving normal or near‐normal motor milestones. Although PI.1 has had oscillating, low‐intensity muscle pain in the legs throughout his life, he did not initially report these symptoms and was regarded as asymptomatic until the onset of the disorder in the children. Serum CK determinations were within normal limits for this patient.

The family first presented in Craiova, Romania, for a neuromuscular evaluation of PII.1 in 2019 (aged 4) due to intermittent bilateral leg pain and usually exercise‐induced fatigability, elevated serum CK at 6,390 U/L, and recurrent infectious episodes. The patient had been treated with corticosteroids, which reduced CK levels to approximately 400 U/L, though this intervention had no effect on the myalgias. Additionally, treatment with methotrexate was associated with a presumed increase in CK levels. The symptomatology persisted during further examinations in Créteil and London, with the patient also starting to experience abdominal muscle spasms increasing in frequency, unaccompanied by any digestive issues. The clinical tableau also included laxity of knees and distal interphalangeal joints and hip and Achilles tendon tightness. There was no evidence of motor deficit, muscle atrophy, muscle pain on palpation, Gowers′ sign, dyspnea, myoglobinuria, or neurological issues, as the patient remained physically active and participated in sports. CK levels continued to be elevated and fluctuating, with peak values exceeding 10,000 U/L; other investigations demonstrated normal myositis and anti‐DNA antibody profiles, normal acid maltase values, and unremarkable plasma amino acids or carnitine profile variations. Cardiac, respiratory, EMG, and leg muscle ultrasound examinations were normal. Lower limb muscle MRI showed no significant abnormalities.

PII.2 was born with a patent *foramen ovale* and exhibited a normal development, aside from a slight delay in independent walking acquisition (18 months of age). His symptomatology resembled PII.1, with muscle pain in the lower limbs, fatigability, joint laxity, and mild hip weakness. Of the three brothers, he is considered more affected, as he experiences fatigue more rapidly and has important leg pain that can awaken him during the night. Also, he experienced chest pain episodes with difficulty in breathing, needing emergency room interventions. The highest value of serum CK was above 5,400 U/L, and a myositis and anti‐DNA antibody panel came back negative. A muscle ultrasound examination was normal, as were cardiorespiratory investigations; however, an EMG test showed clear myopathic features.

Apart from the presence of myalgias primarily affecting the feet and heels, occasionally at night, and joint laxity, the neuromuscular examination of PII.3 was normal. CK levels were elevated, exceeding 5,800 U/L. Plasma amino acid levels were normal, and he had a mild carnitine profile alteration.

Supporting Information 1: Table S1 summarizes the main characteristics of the four patients.

### 3.2. Myopathology

**Figure 2:**
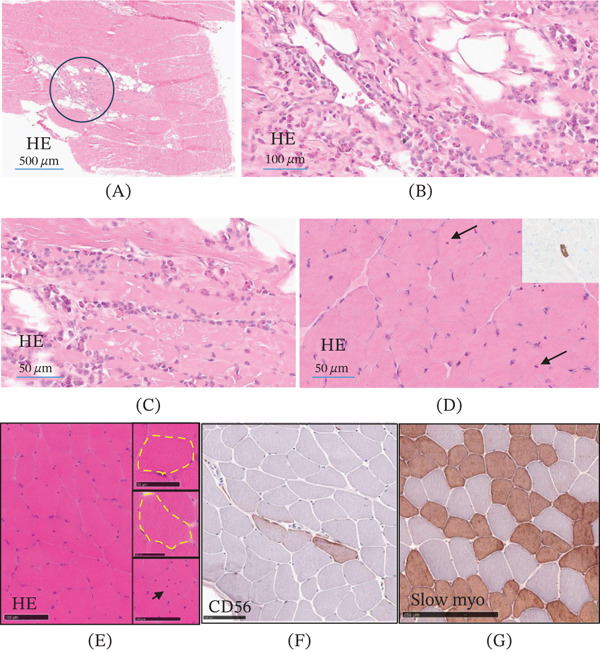
Myopathology. Patient II.1: (A) Hematoxylin and eosin‐stained sections from the left gastrocnemius biopsy at 4 years and 9 months of age showed several well‐populated fascicles comprising predominantly longitudinally sectioned myofibers. There was a single prominent focus of inflammation centered on the perimysium (circle) with a mild increase in perimysial fat. (B) The inflammatory infiltrate was rich in eosinophils and diffusely infiltrated the perimysial connective tissue and (C) surrounding necrotic myofibers. Patient II.2: (D) Hematoxylin and eosin‐stained sections from the left quadriceps biopsy taken at 5 years and 2 months of age showed compact, well‐populated myofascicles. Few fibers (< 1%) showed internal (single, central) nuclei and a rare regenerating fiber expressing embryonic/developmental myosin (inset). Patient I.1: (E) Hematoxylin and eosin‐stained sections showing fiber size diameter variation (yellow dotted lines) and multiple nuclear internalizations (black arrow). (F) CD56 positivity showing regenerating muscle fibers (0.55% of total fibers). (G) Slow myosin staining showing fiber size diameter variation and Type 1 fiber atrophy. Note: Patient II.1: no frozen tissue was available for immunohistochemical assessment. Patient II.2: immunolabeling for a broad panel of dystrophy‐associated markers was normal. Immunolabeling for alpha‐dystroglycan was deemed unreliable due to suboptimal staining in the normal control muscle.

PII.1 muscle biopsy, performed in Romania and re‐examined in France and the United Kingdom by two of the authors (E.M. and R.P.), showed well‐populated fascicles, mild myopathic fiber size variation, and few internalized nuclei, as well as a single focus of eosinophil‐rich mononuclear inflammation centered on the perimysium and extending into the necrotic myofibers in the immediate vicinity, mimicking focal eosinophilic myositis (Figure 2A, B, C). Outside of this focus, there was no significant inflammation, myonecrosis, or regeneration.

Patient II.2 muscle biopsy images revealed mild myopathic changes, including mild fiber size variation, and a few internalized nuclei (Figure 2D). Immunostaining for developmental/embryonic myosin highlighted a rare regenerating fiber (Figure 2D inset).

Patient I.1 (father) is the last member of the family to undergo a muscle biopsy, with the results showing multiple nuclear internalizations, scattered regenerating fibers, and Type I fiber atrophy (Figure 2E,F,G).

### 3.3. Genetics

Initial genetic investigations included a negative *DMD* gene analysis for PII.1 and PII.2, a negative family (mother, father, and three sons) rhabdomyolysis/metabolic myopathy panel, and a WES analysis for PII.2, which identified a heterozygous variant of uncertain significance (VUS) in *POMT1*—c.1859G>A, p.(Arg620Gln). A further CMYO/muscular dystrophy panel, which included all the family members, revealed heterozygous VUS in *DNM2*—c.58C>T, p.(Phe20Leu) (PII.1 and PII.3); *RYR3*—c.2684G>A, p.(Arg895Gln) (mother and PII.2); and *DAG1*—c.887G>A, p.(Gly296Asp) (PI.1, PII.1, PII.2, and PII.3). The presence of the heterozygous *DAG1* Exon 3 variant in PII.1 was also confirmed by raw‐data reanalysis from an independent muscular dystrophy panel and WES.

The heterozygous missense *DAG1* variant, NM_004393.6:c.887G>A, NP_004384.5:p.(Gly296Asp), results in the substitution of a well‐conserved mammal glycine with aspartic acid in the alpha‐dystroglycan subunit. The variant is absent from major aggregation databases (gnomAD v4.1.0/ExAC), as well as from ClinVar. The CADD Phred score is 27.00, whereas missense predictors (SIFT, PolyPhen 2, Fathmm‐XF, AlphaMissense, REVEL, ClinPred, MetaSVM, MetaLR, and Mistic) suggest a deleterious effect [22].

### 3.4. Alpha‐Dystroglycan Glycosylation Studies

**Figure 3:**
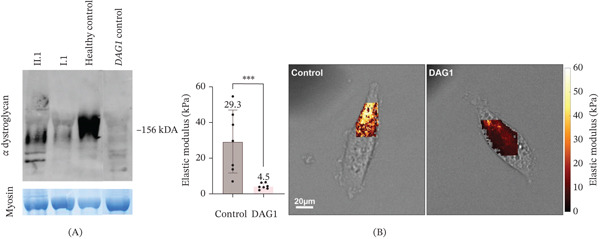
(A) Western blot. Patient I.1: Western blot analysis showing reduced alpha‐dystroglycan expression in Patient I.1 and Patient II.1 compared to a healthy control. Alpha‐dystroglycan expression is absent in *DAG1* control (biallelic variants). (B) Atomic force microscopy (AFM)–based mechanical characterization of DAG1‐deficient myoblasts. Left panel: bar plot showing the mean elastic modulus (±SD) of control and Patient I.1‐derived myoblasts (*n* = 7 per group; Mann–Whitney *U* test, *p* = 0.0006). Patient I.1 myoblasts show a markedly reduced elastic modulus compared to controls (4.5 ± 1.6 vs. 29.3 ± 17.6 kPa) ***p < 0.001. Right panels: representative AFM elasticity maps overlaid on phase‐contrast images of a control myoblast (left) and a Patient I.1‐derived myoblast (right), illustrating the approximately 85% decrease in elastic modulus. Color scale indicates elastic modulus values from 0 to 60 kPa. Scale bar: 20 *μ*m.

A reduced alpha‐dystroglycan glycosylation status for PI.1 and PII.1 was highlighted through Western blot (Figure 3A) and immunohistochemistry studies (not shown). Additionally, RNA‐seq performed in PII.1 did not reveal any abnormal transcripts for dystroglycanopathy genes, thus excluding splicing–altering intronic variants.

### 3.5. Biomechanical Characterization of Myoblasts

AFM‐based biomechanical measurements revealed a marked alteration in the mechanical properties of *DAG1*‐deficient myoblasts (Figure 3B). The Mann–Whitney test demonstrated a highly significant reduction in cell stiffness in patient‐derived myoblasts compared to controls (mean ± SD: 4.48 ± 1.62 vs. 29.35 ± 17.61 kPa; *p* = 0.0006), with a complete separation of the data distributions (Mann–Whitney *U* = 0). This approximately 6.5‐fold decrease in stiffness reflects a drastic shift in cytoskeletal responsiveness and indicates a loss of the structural integrity required for normal muscle cell function.

## 4. Discussion

Recent advances in next‐generation sequencing have allowed the identification of an important number of genes associated with neuromuscular disorders and particularly with LGMDs [5]. Among these, *DAG1*, linked with LGMD R16, is one of the most rarely encountered genes, with only several publications describing patients with biallelic variants, including missense mutations [11, 12, 23, 24]. Furthermore, heterozygous truncating *DAG1* variants have been shown to segregate along with high CK levels or a mild neuromuscular phenotype in five families [15, 16]. Characteristics of previously reported patients with heterozygous *DAG1* variants are available in Supporting Information 2: Table S2. Also, one patient with a heterozygous 2 Mb deletion encompassing *DAG1* has been reported, presenting mild myopathic features, facial hypotonia, oral–motor dyspraxia, learning difficulties, high CK levels, and white matter abnormalities [25]. To date, no heterozygous missense *DAG1* variants have been associated with a neuromuscular phenotype.

All previously known patients with heterozygous *DAG1* variants had elevated CK levels ranging from just over 200 U/L to several thousand units per liter [15, 16], making PI.1 the only patient reported so far with a normal CK level. However, we believe it is possible that, during his earlier years, serum CK levels might have been elevated. Among 16 reported patients from five families, 12 exhibited incidental hyperCKemia; overall, the described clinical features consisted of myalgias, muscle cramps, exercise intolerance, lower girdle weakness, and hyporeflexia, a phenotype broadly similar to the one in our patients. The main reason for evaluation in our family was represented by myalgias, whereas in other patients, the most common initial finding was an incidental increased CK level. Regarding patients with LGMD R16 (homozygous *DAG1* variants), although clinical presentations can vary and severe forms are possible [1, 11], mild forms with late onset or asymptomatic hyperCKemia have been reported [23, 24].

In terms of muscle biopsy aspects, the findings in our patients are consistent with those in other *DAG1* haploinsufficiency patients, who present mild fiber size variability, central nuclei, and degenerative fibers [15]. Also, from a muscle MRI perspective, there were no abnormalities in four previously examined patients, except for one patient showing diffusely increased muscle bulk, as was the case for PII.1 [15]. This is somehow expected, taking into account the mild phenotypes exhibited.

The diagnosis odyssey of this family has encountered several obstacles, with the eosinophil‐rich inflammatory features present in the muscle biopsy of PII.1 raising the question of a focal eosinophilic myositis diagnosis. However, this hypothesis was considered unlikely given the lack of response to corticosteroid therapy and the emergence of similar symptoms in the other children, pointing to a common genetic cause. The identification of a *DAG1* variant in all affected individuals was insufficient initially, as it was a VUS, and a second variant could not be identified in order to support an autosomal recessive LGMD R16 diagnosis. Since patients with mild phenotypes and heterozygous *DAG1* variants were later reported [15, 16] and pathological findings were also observed in the muscle biopsy of PI.1 (suggesting a segregation of the variant along with the phenotype), the *DAG1* variant was viewed as a possible cause. The results from the functional studies showing reduced alpha‐dystroglycan glycosylation in two patients provided additional evidence supporting the pathogenic character of this variant, as well as the diagnosis of a mild–moderate, autosomal dominant LGMD R16. Considering all the data, the variant is classified as pathogenic based on the following ACMG/AMP criteria: PS3, PM1, PM2, PP1, and PP3.

In the previous publications describing patients with mild phenotypes associated with heterozygous *DAG1* variants [15, 16], all reported variants were either nonsense or frameshift pathogenic mutations. In this family, the reported variant has a missense effect, involving a substitution of glycine with aspartic acid at position 296 of the amino acid chain; this finding could support the pathogenicity of the variant, as the site corresponds to the N‐terminal domain of the alpha‐dystroglycan subunit, essential for glycosylation [7, 24]. Notably, homozygous missense variants resulting in N‐terminal domain substitutions have been known to interfere with the alpha‐dystroglycan glycosylation and receptor function [12, 23].

Cell stiffness in myoblasts is primarily governed by the organization of the cortical actin network and actin stress fibers, anchored to the ECM through focal adhesion complexes [26]. These structures mediate bidirectional mechanotransduction between the intracellular cytoskeleton and the ECM and are principal determinants of myoblast mechanical properties. The dystrophin–glycoprotein complex (DGC) participates in this mechanotransduction framework by connecting the ECM to the intracellular cytoskeleton, providing structural stability to the muscle membrane [27, 28]. Within the DGC, alpha‐dystroglycan bridges extracellular laminin and agrin to beta‐dystroglycan, which in turn connects to the intracellular cytoskeleton through dystrophin [29]. This interaction is strictly dependent on the glycosylation status of alpha‐dystroglycan: hypoglycosylation abolishes its binding to ECM ligands, disrupting the mechanical continuity between the cytoskeleton and the ECM [30, 31].

In the present study, Western blot analysis revealed a reduced alpha‐dystroglycan glycosylation status in PI.1, consistent with *DAG1* haploinsufficiency. This finding is in line with the biochemical defects reported by Traverso et al. in patients harboring heterozygous truncating variants in *DAG1*, where a markedly reduced expression of the dystroglycan complex was confirmed on muscle samples [15]. Impaired glycosylation of alpha‐dystroglycan disrupts its binding to ECM ligands, weakening the structural continuity between the ECM and the intracellular cytoskeleton [30, 31]. In muscle precursor cells, this is expected to compromise actin cytoskeleton organization and reduce cell stiffness, as reflected by the reported AFM measurements.

This mechanical incompetence is expected to impair critical cellular functions that depend on cytoskeletal tension, including directional migration, myoblast alignment, and fusion into myotubes—processes collectively required for effective muscle regeneration [32, 33]. Notably, the biophysical deficit observed by AFM contrasts with the mild clinical presentation of the patients, consistent with the concept of a genetically transitional disease, in which *DAG1* haploinsufficiency is sufficient to affect muscle structure at the cellular level while phenotypic expressivity remains modulated by the broader genetic background [15, 34]. Taken together, the AFM data and the biochemical evidence of alpha‐dystroglycan hypoglycosylation provide converging biophysical and molecular support for the conclusion that heterozygous *DAG1* missense variants can compromise the mechanical stability of muscle precursor cells, offering a mechanistic basis for the observed myopathic phenotype despite a mild clinical presentation.

Interestingly, PII.2 seems to have a slightly more severe phenotype compared to his siblings and father, with leg pain during the night, whereas his muscle biopsy demonstrated milder changes in comparison with PII.1, and his CK levels have not reached the same elevations observed in his brothers. Since pain is a highly subjective symptom, this variation might not be relevant; however, the possibility of other modifiers cannot be entirely excluded. In this regard, two variants of interest are harbored only by PII.2, in *POMT1* (c.1859G>A, p.(Arg620Gln)) and *RYR3* (c.2684G>A, p.(Arg895Gln)), respectively. *POMT1* is associated with another alpha‐dystroglycanopathy, LGMD R11. This condition is autosomal recessive, and myopathological features observed in affected patients could overlap with the ones demonstrated in PII.2 [35]. Regarding the *RYR3* variant, this gene is linked to a nemaline CMYO, and although some clinical features could correspond to our patient′s phenotype, the muscle biopsy did not highlight any rods [36]. As for *DNM2*, since this gene is associated with an autosomal dominant centronuclear myopathy [37], a phenotype not in keeping with the clinical and myopathological features presented by PII.1 and PII.3, who harbor the variant c.58C>T, p.(Phe20Leu), we believe this variant is unlikely to be a contributor or modifier.

To conclude, the data obtained from this family supports the hypothesis that heterozygous missense *DAG1* variants can affect alpha‐dystroglycan glycosylation and lead to a mild–moderate muscular phenotype and that patients could also have normal CK levels. Given the presentation, it is plausible that such patients remain underdiagnosed, and screening strategies involving serum CK determination could help in this regard. Since these patients seem to have mild phenotypes and acknowledging a dominant form of the disease would further complicate the classification of LGMDs, we propose the term “symptomatic carrier of LGMD R16” for individuals with such profiles.

## Author Contributions

Conceptualization: Edoardo Malfatti, Adnan Manzur, and Amelia Dobrescu. Data curation and formal analysis: Alexandru Caramizaru, Federica Trentin, Andreea Dumitrescu, Luca Sali, Alexandra Bastian, Hane Lee, Camille Verebi, Juliette Nectoux, France Leturcq, Rahul Phadke, and Redouane Fodil (AFM data analysis). Funding acquisition: Edoardo Malfatti, Homa Tajsharghi, France Leturcq, Adnan Manzur, and Amelia Dobrescu. Investigation: all authors. Methodology: Edoardo Malfatti, Adnan Manzur, Redouane Fodil (AFM measurements and biomechanical approach), and Amelia Dobrescu. Project administration: Edoardo Malfatti. Resources: all authors. Software: Federica Trentin, Hane Lee, Camille Verebi, and Juliette Nectoux. Supervision: Edoardo Malfatti, Adnan Manzur, and Amelia Dobrescu. Visualization: Alexandru Caramizaru, Federica Trentin, Camille Verebi, Juliette Nectoux, Rahul Phadke, and Redouane Fodil (Figure 3B). Writing—original draft preparation: Edoardo Malfatti and Alexandru Caramizaru. Writing—review and editing: Edoardo Malfatti, Alexandra Bastian, Hane Lee, Homa Tajsharghi, France Leturcq, Anna Sarkozy, Adnan Manzur, Redouane Fodil, and Amelia Dobrescu. Edoardo Malfatti and Alexandru Caramizaru contributed equally to this work.

## Funding

This study was funded by the 3billion, End the Diagnostic Odyssey—Prize granted to Prof. H. Tajsharghi.

## Ethics Statement

Patient consent for participation and publication was obtained. The study was designed according to the Declaration of Helsinki, and it has been approved by the ethical committees of the involved institutions and, for France, by the Comité de Protection Est IV DC‐2012‐1693.

## Conflicts of Interest

The authors declare no conflicts of interest.

## Supporting Information

Additional supporting information can be found online in the Supporting Information section.

## Supporting information


**Supporting Information 1** Table S1: Main characteristics (clinical, myopathology, genetic, and functional data) of the four patients.

**Supporting Information 2** Table S2: Comparison with previously reported patients with heterozygous *DAG1* variants.

## Data Availability

All data generated or analyzed during this study are included in this published article.
