# Supplementary material for: Autosomal Dominant Missense DAG1 Variant Linked to Mild–Moderate LGMD R16
Source: Hum Mutat. 2026 Jul 4;2026:7451586. doi: 10.1155/humu/7451586 (PMC13332352; doi:10.1155/humu/7451586)
Supplement: Supplementary file 1 — Supporting Information 1 Table S1: Main characteristics (clinical, myopathology, genetic, and functional data) of the four patients. [file HUMU-2026-7451586-s002.docx]

**Supplementary Table 1. Main characteristics (clinical, myopathology, genetic, functional data) of the four patients.**

|  | **Father (I.1)** | **Eldest son** (**II.1)** | **Middle son (II.2)** | **Youngest son (II.3)** |
| --- | --- | --- | --- | --- |
| **Age (years)** | 42 | 10 | 9 | 6 |
| **Motor development** | normal | normal | normal, mild delay in walking acquisition | normal |
| **Onset** | unknown | 4 years old | 3 years old | 1 year old |
| **Clinic** | intermittent low-intensity leg pain | fatigability, intermittent leg pain (mostly exercise-induced) | fatigability, intermittent leg pain (exercise-induced and at night) | intermittent leg pain (mostly exercise-induced) |
| **CK levels** | normal | increased, fluctuating, highest value over 10,000 U/L, never normal | increased, fluctuating, highest value over 600 U/L, sometimes normal | increased, fluctuating, highest value over 1,400 U/L, never normal |
| **Muscle biopsy** | (left deltoid, aged 41) myopathic changes, nuclei internalizations, type I fibres atrophy | (left gastrocnemius, aged 4) myopathic changes, increased fibre size variability, type 1 fibres predominance, diffuse inflammatory infiltrate, perivascular monocytes and eosinophiles, phagocytosis | (left quadriceps, aged 5) mild myopathic changes, mild fibres size variation, internal nuclei | not performed |
| **Muscle MRI** | not performed | no significant abnormality | not performed | not performed |
| **EMG** | not performed | normal | myopathic pattern | not performed |
| **DAG1 variant NM_004393.6:c.887G>A, NP_004384.5:p.(Gly296Asp)** | present | present | present | present |
| **alpha-dystroglycan glycosylation studies** | decreased | decreased | not performed | not performed |
| **DAG1 RNAseq** | not performed | no abnormal transcripts | not performed | not performed |
| **Atomic Force Microscopy** | significant reduction in myoblast stiffness | not performed | not performed | not performed |
