# Supplementary material for: Autosomal Dominant Missense DAG1 Variant Linked to Mild–Moderate LGMD R16
Source: Hum Mutat. 2026 Jul 4;2026:7451586. doi: 10.1155/humu/7451586 (PMC13332352; doi:10.1155/humu/7451586)
Supplement: Supplementary file 2 — Supporting Information 2 Table S2: Comparison with previously reported patients with heterozygous DAG1 variants. [file HUMU-2026-7451586-s001.docx]

**Supplementary Table 2. Comparison with previously reported patients with heterozygous *DAG1* variants.**

| **Patient** | **Onset / First evaluation** | **Clinic** | **CK levels** | **Muscle biopsy** | **Muscle**  **MRI** | **DAG1 variant** | **alpha-dystroglycan** |
| --- | --- | --- | --- | --- | --- | --- | --- |
| **I.1 (current study)** | 42 y | intermittent low-intensity leg pain | normal | myopathic changes, nuclei internalizations, type I fibres atrophy | not performed | c.887G>A, missense | decreased |
| **II.1 (current study)** | 4 y | fatigability, intermittent leg pain (mostly exercise-induced) | increased, fluctuating, highest value over 10,000 U/L, never normal | myopathic changes, increased fibre size variability, type 1 fibres predominance, diffuse inflammatory infiltrate, perivascular monocytes and eosinophiles, phagocytosis | no significant abnormality | c.887G>A, missense | decreased |
| **II.2 (current study)** | 3 y | mild delay in walking acquisition, fatigability, intermittent leg pain (exercise-induced and at night) | increased, fluctuating, highest value over 600 U/L, sometimes normal | mild myopathic changes, mild fibres size variation, internal nuclei | not performed | c.887G>A, missense | not performed |
| **II.3 (current study)** | 1 y | intermittent leg pain (mostly exercise-induced) | increased, fluctuating, highest value over 1,400 U/L, never normal | not performed | not performed | c.887G>A, missense | not performed |
| **I-1(Fan et al. [1])** | unknown | aymptomatic hyperCKemia | elevated | not performed | not performed | c.930delC:p.R311Gfs*70, frameshift | not performed |
| **II-1 (Fan et al. [1])** | 10 y | aymptomatic hyperCKemia | elevated | not performed | not performed | c.930delC:p.R311Gfs*70, frameshift | not performed |
| **II-2 (Fan et al. [1])** | 11 y | aymptomatic hyperCKemia | elevated | not performed | not performed | c.930delC:p.R311Gfs*70, frameshift | not performed |
| **II-3 (Fan et al. [1])** | 14 y | aymptomatic hyperCKemia | elevated | not performed | not performed | c.930delC:p.R311Gfs*70, frameshift | not performed |
| **1 (Traverso et al. [2])** | 10 y | incidental hyperCKemia, reduced deep tendon reflexes | highest value 1,832 U/L | not performed | not performed | c.832delG | not performed |
| **2 (Traverso et al. [2])** | 30 y | muscle cramps | highest value 2,240 U/L | mild fiber size variability | normal | c.832delG | decreased |
| **3 (Traverso et al. [2])** | 8 y | incidental hyperCKemia, exercise intolerance, reduced deep tendon reflexes | highest value1,445 U/L | not performed | not performed | c.1918C>T | not performed |
| **4 (Traverso et al. [2])** | 29 y | myalgia | highest value 450 U/L | not performed | not performed | c.1918C>T | not performed |
| **5 (Traverso et al. [2])** | 28 y | myalgia | highest value 1,383 U/L | mild fiber size variability, central nuclei, 1 degenerative fiber | \|  \| diffuse increase of muscle bulk without fat or fibrotic infiltration \| \| --- \| --- \| | c.1925_1926delGT | decreased |
| **6 (Traverso et al. [2])** | 39 y | exercise intolerance, lower girdle weakness (MRC 4) | highest value 598 U/L | not performed | normal | c.71_72delTG | not performed |
| **7 (Traverso et al. [2])** | 16 y | incidental hyperCKemia | highest value 1,300 U/L | not performed | normal | c.2167delC | not performed |
| **8 (Traverso et al. [2])** | 42 y | incidental hyperCKemia | highest value 911 U/L | not performed | not performed | c.164C>G | not performed |
| **9 (Traverso et al. [2])** | 61 y | incidental hyperCKemia | highest value 304 U/L | not performed | not performed | c.164C>G | not performed |
| **10 (Traverso et al. [2])** | 73 y | incidental hyperCKemia | highest value 270 U/L | not performed | not performed | c.164C>G | not performed |
| **11 (Traverso et al. [2])** | 31 y | incidental hyperCKemia | highest value 988 U/L | not performed | not performed | c.164C>G | not performed |
| **12 (Traverso et al. [2])** | 19 y | incidental hyperCKemia | highest value 2,800 U/L | mild fiber size variaility | not performed | c.330G>A | decreased |

**References**

1. Fan, L., et al., *A novel 1-bp deletion variant in DAG1 in Japanese familial asymptomatic hyper-CK-emia.* Hum Genome Var, 2022. **9**(1): p. 4.

2. Traverso, M., et al., *DAG1 haploinsufficiency is associated with sporadic and familial isolated or pauci-symptomatic hyperCKemia.* Eur J Hum Genet, 2024. **32**(3): p. 342-349.
